# MicroRNA-223 Suppresses IL-1β and TNF-α Production in Gouty Inflammation by Targeting the NLRP3 Inflammasome

**DOI:** 10.3389/fphar.2021.637415

**Published:** 2021-04-16

**Authors:** Quan-Bo Zhang, Dan Zhu, Fei Dai, Yu-Qin Huang, Jian-Xiong Zheng, Yi-Ping Tang, Zeng-Rong Dong, Xia Liao, Yu-Feng Qing

**Affiliations:** ^1^ Research Center of Hyperuricemia and Gout, Affiliated Hospital of North Sichuan Medical College, North Sichuan Medical College, Nanchong, China; ^2^ Department of Geriatrics, Affiliated Hospital of North Sichuan Medical College, North Sichuan Medical College, Nanchong, China; ^3^ Department of Rheumatology and Immunology, Affiliated Hospital of North Sichuan Medical College, Nanchong, China; ^4^ Department of Rheumatology and Immunology, Daping Hospital, Army Medical University, Chongqing, China

**Keywords:** microrna-223, gout, inflammation, cytokines, inflammasome, NLRP3

## Abstract

**Introduction:** MicroRNA-223 (MiR-223) serves as an important regulator of inflammatory and immune responses and is implicated in several auto-inflammatory disorders. Here, we measured miR-223 expression in acute and intercritical gout patients, after which we used RAW264.7 macrophages transfected with a miR-223 mimic/inhibitor to determine the function of miR-223 in monosodium urate (MSU)-induced gouty inflammation.

**Methods and Results:** MiR-223 was detected among 122 acute gout patients (AG), 118 intercritical gout patients (IG), and 125 healthy subjects (HC). RAW264.7 macrophages were cultured and treated with MSU. Over-expression or under-expression of miR-223 was inducted in RAW264.7 macrophages to investigate the function of miR-223. Real-time quantitative PCR, ELISA and western blotting were used to determine the expression levels of miR-223, cytokines and the NLRP3 inflammasome (NLRP3, ASC, and caspase-1). MiR-223 expression was significantly decreased in the AG group in comparison with the IG and HC groups (*p* < 0.001, respectively). Up-regulated expression of miR-223 was observed after acute gout remission in comparison with that observed during gout flares in 30 paired cases (*p* < 0.001). The abundance of the NLRP3 inflammasome and cytokines was significantly increased after RAW264.7 macrophages were treated with MSU (*p* < 0.01, respectively), while that of miR-223 was significantly reduced (*p* < 0.01). Up-regulation of miR-223 decreased the concentrations of IL-1β and TNF-α, as well as the NLRP3 inflammasome expression (p < 0.01, respectively), while IL-37 and TGF-β1 levels were unchanged (*p* > 0.05, respectively). Under-expression of miR-223 increased the concentrations of IL-1β and TNF-α, as well as NLRP3 inflammasome expression (*p* < 0.01, respectively), while IL-37 and TGF-β1 levels were not influenced (*p* > 0.05, respectively).

**Conclusion:** These findings suggest that miR-223 provides negative feedback regulation of the development of gouty inflammation by suppressing production of IL-1β and TNF-α, but not by regulating IL-37 and TGF-β1. Moreover, miR-223 regulates cytokine production by targeting the NLRP3 inflammasome.

## Introduction

Gout, one of the most common forms of autoinflammatory arthritis in humans, is characterized by elevated urate and monosodium urate (MSU) crystal deposition in tissues, which leads to arthritis, soft tissue masses (i.e., tophi), nephrolithiasis, and urate nephropathy (Dalbeth et al., 2016). The specific pathogenesis of gout is unclear. Previous studies have demonstrated that an attack of gouty arthritis is triggered by the deposition of MSU crystals in the joint, and MSU crystals are widely recognized as an endogenous danger signal by components of the innate immune system (Martinon et al., 2006).

NACHT-LRR-PYD-containing protein (NLRP3) is a pattern recognition receptor in the innate immune system. NLRP3 inflammasome (NLRP3, apoptosis-associated speck-like protein (ASC), and caspase-1) signaling is involved in the pathogenesis of interleukin (IL)-1β-mediated gouty arthritis (Martinon et al., 2006; So and Martinon, 2017; Renaudin et al., 2020). Spontaneous resolution is one of the characteristics that differentiate gout from other arthropathies and auto-inflammatory diseases (Steiger and Harper, 2014). Previous reports suggest that IL-37 and transforming growth factor β1 (TGF-β1) play key roles in limiting inflammation and might be involved in spontaneous remission of gouty arthritis (Lioté et al., 1996; Zeng et al., 2016).

MicroRNAs (miRs) are short non-coding RNAs with a length of approximately 22 nucleotides. Recently, the role of miRs in negatively regulating inflammation in autoimmune diseases and inflammation disorders has received increasing attention (Henshall et al., 2016; Long et al., 2020). miR-223 is expressed in myeloid cells, and miR-223 under-expression is associated with many diseases, including leukemia, hepatitis B, influenza, lymphoma, and inflammation (Gilicze et al., 2014). Previously, it was found that NLRP3 is one of the target genes of miR-223 (Bauernfeind et al., 2012). Recent studies have confirmed that miR-223 can inhibit inflammation by targeting NLRP3, thereby suppressing inflammasome activation and pyroptosis in T pallidum-infected endothelial cells (Long et al., 2020), limiting inflammation in human dental pulp fibroblasts (Wang et al., 2020), relieving spinal cord injury (Zhang et al., 2020), and modulating dendritic cell function to ameliorates autoimmune myocarditis (Zhou et al., 2017). However, the status of miR-223 expression and the potential role of miR-223 in gout have not been reported.

To date, no relevant studies have reported the expression levels of miR-223 in gout patients or assessed whether miR-223 participates in negatively regulating gouty inflammation via regulating cytokines (such as IL-1β, tumor necrosis factor (TNF)-α, IL-37 and TGF-β1) by targeting the NLRP3 inflammasome. Therefore, the aim of our study was to measure miR-223 expression in peripheral blood mononuclear cells (PBMCs) from patients with acute gout (AG), patients with intercritical gout (IG) and healthy subjects (HC). In addition, RAW264.7 macrophages were transfected with a miR-223 mimic or inhibitor to determine the function of miR-223 in MSU-induced gouty inflammation.

## Materials and Methods

### Patients and Controls

A cross-sectional study was carried out the Affiliated Hospital of North Sichuan Medical College from June 2019 to December 2020. Two hundred and forty consecutive male patients with primary gout were enrolled in the study. The classification of gout fulfilled the 1977 American Rheumatism Association (now the American College of Rheumatology (ACR)) preliminary criteria for classification of acute arthritis of primary gout, as well as the 2015 ACR/European League Against Rheumatism (EULAR) gout classification criteria (Wallace et al., 1977; Neogi et al., 2015). All gout patients had no history of cancer, hematopathy, nephropathy, infection or other autoimmune diseases. Gout patients were divided into an acute gout (AG) group (including 122 patients with acute gout attacks) and an intercritical gout (IG) group (including 118 patients) based on whether patients presented with gout flares or not. In detail, gout patients with symptoms such as joint swelling, heat and pain within 3 days were acute group; gout patients had no joint symptoms for at least 2 weeks, and inflammatory indicators such as erythrocyte sedimentation rate (ESR < 21 mm/h), high-sensitivity C-reactive protein (CRP < 9 mg/L), white blood cell count (3.50–9.50 × 10^9^/L) in the normal range were intercritical group. Healthy controls (HC, 125 age-matched men) with no hyperuricemia, no metabolic syndrome, and no other chronic diseases were recruited from the Physical Examination Center of the Affiliated Hospital of North Sichuan Medical College. Peripheral blood samples were obtained from patients with gout and healthy controls. Serum samples were stored at −80°C until cytokines levels were determined. PBMCs were isolated from patients and healthy controls using Ficoll-Paque PLUS (GE Healthcare, Piscataway, NJ, United States) according to the manufacturer’s instructions. Written informed consent was obtained from all of the enrolled participants. Ethical approval was obtained for the study from the relevant ethics committees.

### Laboratory Examination of Regulatory Parameters

Clinical laboratory evaluations of serum uric acid (sUA) level, blood glucose (GLU) level, serum globulin level, inflammation and lipid metabolism indicators were performed. The inflammation indicators estimated included erythrocyte sedimentation rate (ESR), high-sensitivity C-reactive protein (CRP), leukocyte counts, lymphocyte counts, neutrophil cell counts, monocyte counts, and the lipid metabolism indicators include plasma total cholesterol (TC), triglycerides (TG). All of the measurements were carried out by the Clinical Laboratory Department of the Affiliated Hospital of North Sichuan Medical College.

### Preparation of MSU Crystals

MSU crystals were prepared under pyrogen-free conditions. Briefly, 1 g uric acid (Sigma-Aldrich, St. Louis, MO, United States) was dissolved in 200 ml of boiling water containing 6 ml of 1 N NaOH. The pH value of the final solution was adjusted to 7.2 by adding HCl. The solution was cooled, stirred at room temperature, and stored overnight at 4°C. The precipitate was filtered from the solution and dried under low heat. The crystals were weighed under sterile conditions and suspended in PBS at a concentration of 25 mg/ml (Zhang et al., 2018).

### RAW264.7 Macrophage Culture and Cell Stimulation

RAW264.7 macrophage cells were purchased from the American Type Culture Collection (Rockville, MD, United States). Cells were cultured in DMEM supplemented with 10% FBS and antibiotics (100 U/ml penicillin and 100 U/ml streptomycin) at 37°C in a humidified incubator with an atmosphere containing 5% CO_2_. The culture medium was refreshed once every 2 days. The cells were seeded into 6-well plates at a density of 6 × 10^5^ cells/well. After 12 h, the plated cells in the experimental group were stimulated with MSU (200 μg/ml), whereas the control group was treated with an equal volume of the culture medium. The culture supernatants and cells were collected at 0, 3, 6, and 12 h after stimulation.

### Cell Transfection With a miR-223 Mimic or Inhibitor

RAW264.7 macrophages were transfected in 6-well plates at a density of 1 × 10^5^ cells/well using Lipofectamine 2000 reagent (Invitrogen) according to the manufacturer’s protocol. The cells were transfected with 80 nM (final concentration) of synthetic mature miR-223 molecule (miR-223 mimic), an antagomir antisense to mature miR-223 (miR-223 inhibitor), or a scrambled control transcript serving as a negative control (Ribobio, Ltd., China) 24 h prior to stimulation with MSU (200 μg/ml). Twelve hours after stimulation with MSU, the expression levels of miR-223, NLRP3, ASC, caspase-1, IL-1β, TNF-α, IL-37, and TGF-β1 were measured. In separate experiments, RAW264.7 macrophages were transfected and treated as described above, and their apoptotic status was assayed 48 h after transfection and 24 h after stimulation.

### RNA Isolation and Real-Time Quantitative Polymerase Chain Reaction

Total RNA was extracted from RAW264.7 macrophages and the PBMCs of gout patients and healthy controls patients using TRI-zol reagent (TaKaRa, Japan). Reverse transcription was performed using the PrimeScript® RT reagent kit. The cDNAs of miR-223 and U6 were synthesized using stem-loop reverse transcription primers (miR-223: 5′-GTC​GTA​TCC​AGT​GCA​GGG​TCC​GAG​GTA​TTC​GCA​CTG​GAT​ACG​ACT​GGG​GT-3′; U6: 5′-AAC​GCT​TCA​CGA​ATT​TGC​GT-3′). Following pre-amplification, gene expression was assessed on a 7900HT Fast RT-qPCR System (Applied Biosystems), using the manufacturer’s recommended protocol. SYBR Green II was used to compare the relative expression levels of specific mRNAs (including IL-1β, NLRP3, ASC, and caspase1 mRNAs). RT-qPCR was performed to obtain a mean CT value for each sample. The CT values of the samples were compared using the 2^−ΔΔCT^ method, and β-actin expression was used as an internal reference. The primer sequences are shown in Table 1.

**TABLE 1:** Real-time fluorescent quantitative PCR primers.

	Forward sequence (5–3′)	Reverse sequence (5–3′)
MiR-223	TGG​CTG​TCA​GTT​TGC​AAA​T	GTGCAGGGTCCGAGGT
U6	CTCGCTTCGGCAGCACA	AAC​GCT​TCA​CGA​ATT​TGC​GT
IL-1β	GGA​CAG​CCC​AGG​TCA​AAG​G	AGT​TGA​CGG​ACC​CCA​AAA​GAT
NLRP3	CTG​GAC​CAC​CCC​CTG​CTG​AGA	GGA​AGA​AGC​CCT​TGC​ACC​CCT​CA
ASC	TCA​CAG​AAG​TGG​ACG​GAG​TG	TGT​CTT​GGC​TGG​TGG​TCT​CT
Caspase-1	CGT​GGA​GAG​AAA​CAA​GGA​GTG	AAT​GAA​AAG​TGA​GCC​CCT​GAC
β-actin	CAT​GTA​CGT​TGC​TAT​CCA​GGC	CTC​CTT​AAT​GTC​ACG​CAC​GAT

### Apoptosis Detection

Apoptosis of RAW264.7 macrophages was measured after transfection with a miR-223 mimic, a miR-223 inhibitor, or a scrambled control transcript for 48 h at 37°C. Apoptosis was measured via flow cytometric detection of annexin V binding and propidium iodide (PI) staining (annexin VFITC) according to the manufacturer’s instructions.

### Cytokine Measurement

After RAW264.7 murine macrophages were stimulated with MSU, supernatants were collected and secreted cytokines (including IL-1β, TNF-α, IL-37, and TGF-β1) were detected using ELISA according to the manufacturer’s recommended protocol.

### Western Blot Analysis

Cells were disrupted in lysis buffer, after which the protein concentration was measured using a BCA Protein Assay Kit (Thermo Fisher Scientific, Rockford, IL, United States). The samples were separated via 10% SDS-PAGE and electro-transferred at 90 V to an Immun-Blot PVDF membrane for 2 h. The membranes were blocked in I-Block™ Protein-Based Blocking Reagent for 30 min at room temperature and incubated with primary antibodies (against NLRP3, ASC, caspase-1, IL-1β, and GAPDH) overnight at 4°C. The blots were washed extensively in TBST and incubated with secondary antibodies for 2 h at room temperature. The signal was detected using an enhanced chemiluminescence (ECL) kit (Amersham Pharmacia Biotech, Piscataway, NJ, United States). All antibodies were purchased from Santa Cruz Biotechnology (Santa Cruz Biotechnology, Santa Cruz, CA, United States).

### Statistical Analysis

Statistical analysis was performed with SPSS 17 (IBM Co., Armonk, NY, United States). Comparisons among the AG patients, IG patients and healthy control subjects were mainly performed with one-way analysis of variance (ANOVA) in conjunction with the Bonferroni method for multiple comparisons. Student’s *t*-test with paired samples was used to assess differences in miR-223 expression in PBMCs collected during gout flares and after resolution. The gene expression results were analyzed using the 2^−ΔΔCt^ method. Data from 5 individual experiments were pooled for analysis via Student’s *t*-test.

## Results

### Clinical and Laboratory Characteristics of the Study Subjects

The clinical and laboratory data of the subjects are summarized in Table 2. In this study, gout cases were matched to control individuals by age and gender. Significant differences in body mass index (BMI), serum uric acid (sUA) concentration, glucose level, leukocyte cell count, neutrophil cell count, monocyte cell count, triglyceride (TG) level and globulin level were observed among the AG, IG and healthy control groups (*p* < 0.05). The sUA concentrations of the AG and IG patients were much higher than that of the healthy control subjects (*p* < 0.05). The leukocyte cell count, neutrophil cell count, and monocyte cell count of the AG patients were significantly increased in comparison with the corresponding measurements in the IG patients and healthy control subjects, while the BMI, glucose level, and TG level of the AG and IG patients were much higher than those of the healthy control subjects (*p* < 0.05, respectively). The erythrocyte sedimentation rate (ESR) and C-reactive protein (CRP) level of the AG patients were significantly increased in comparison with those of the IG patients (*p* < 0.05).

**TABLE 2:** Clinical and laboratory data of the subjects.

	AG group (*n* = 122)	IG group (*n* = 118)	HC group (*n* = 125)
Age (years)	44.07 ± 13.97	45.69 ± 12.18	46.00 ± 14.54
Gender F/M	0/122	0/118	0/125
BMI (kg/m^2^) ($\bar{x}$ ± SD)	26.36 ± 4.40^a^	25.99 ± 3.55^a^	22.44 ± 2.04
sUA (μmol/L) ($\bar{x}$ ± SD)	490.87 ± 112.24^a^ ^,^ ^b^	429.05 ± 148.37^a^	352.06 ± 43.34
Glucose (mmol/L) ($\bar{x}$ ± SD)	5.72 ± 0.74^a^	5.71 ± 0.97^a^	4.71 ± 0.44
Leukocyte (×10^9^/L) ($\bar{x}$ ± SD)	9.84 ± 2.33^a^ ^,^ ^b^	5.02 ± 0.66	4.88 ± 0.70
Neutrophil cell (×10^9^/L) ($\bar{x}$ ± SD)	6.71 ± 2.24^a^ ^,^ ^b^	2.94 ± 0.73	2.84 ± 0.59
Lymphocyte (×10^9^/L) ($\bar{x}$ ± SD)	2.21 ± 0.72^a^ ^,^ ^b^	1.40 ± 0.55	1.39 ± 0.50
Monocyte (×10^9^/L) ($\bar{x}$ ± SD)	0.61 ± 0.23^a^ ^,^ ^b^	0.31 ± 0.16	0.30 ± 0.09
TG (mmol/L) ($\bar{x}$ ± SD)	2.40 ± 1.27^a^	2.46 ± 1.24^a^	1.49 ± 1.20
TC (mmol/L) ($\bar{x}$ ± SD)	4.72 ± 0.76	4.87 ± 0.80^a^	4.53 ± 0.67
Globulin (g/L) ($\bar{x}$ ± SD)	33.88 ± 4.25^a^ ^,^ ^b^	32.21 ± 2.85^a^	30.28 ± 2.95
ESR (mm/h) ($\bar{x}$ ± SD)	24.56 ± 21.98^b^	7.86 ± 6.89	—
CRP (mg/L) ($\bar{x}$ ± SD)	19.12 ± 22.93^b^	2.47 ± 2.34	—

AG, acute gout; BMI, body mass index; CRP, reactive protein; ESR, erythrocyte sedimentation rate; HC, healthy control subjects; IG, intercritical gout; sUA, serum uric acid; TG, triglycerides; TC, total cholesterol.

^a^ *p* < 0.05 (in comparison with the HC group).

^b^ *p* < 0.05 (in comparison with the IG group).

### Expression of miR-223 in PBMCs Among the AG, IG, and HC Groups

**FIGURE 1:**
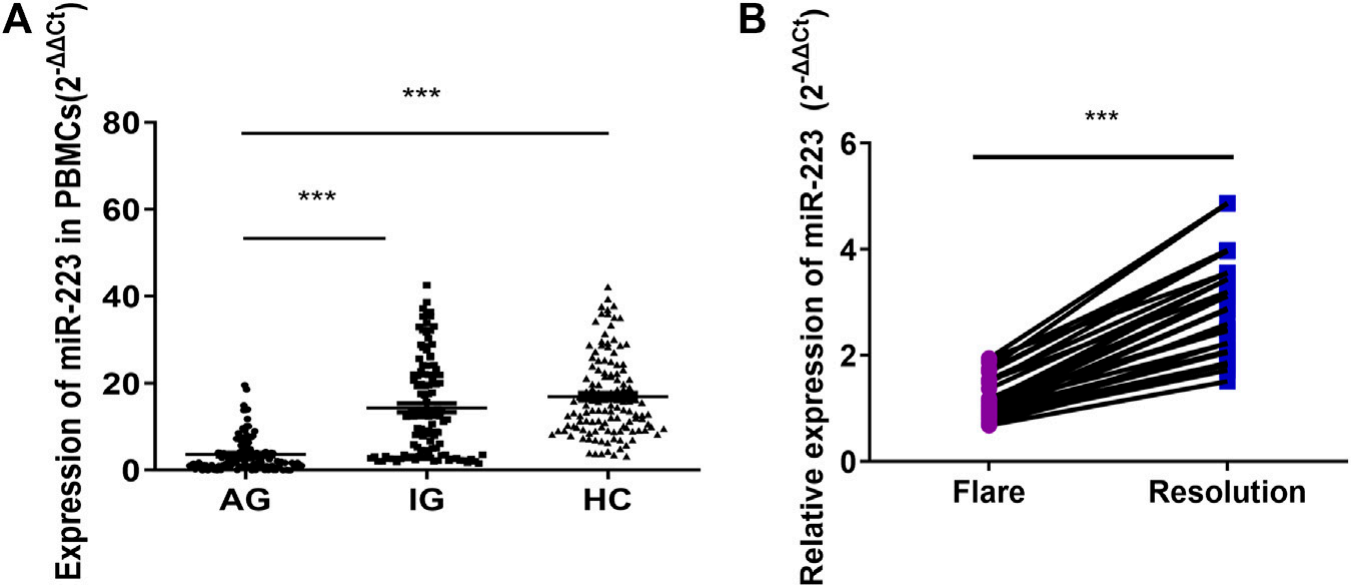
Expression of miR-223 in gout patients. **(A)** Expression of miR-223 in peripheral blood mononuclear cells from patients with acute gout (*n* = 122), patients with intercritical gout (*n* = 118) and healthy control subjects (*n* = 125), one-way ANOVA F = 83.811, *p* < 0.001. ***p < 0.001 for the Bonferroni test for the comparison between groups. **(B)** Relative expression of miR-223 in patients with gout flare and after resolution. Paired *t*-test (*n* = 30). t = −15.649, *p* < 0.001.

The miR-223 level of PBMCs from the AG group was significantly lower than those of the IG and HC groups (*p* < 0.001; Figure 1A), while no difference was observed between the IG and HC groups (*p* > 0.05; Figure 1A). The miR-223 expression level of PBMCs did not differ according to diabetes status, serum urate levels, tophus status or disease duration (data not shown). Negative correlations between miR-223 expression and leukocyte and neutrophil cell counts were observed in acute gout patients (r_s_ = −0.679, −0.612, *p* < 0.001, respectively). Interestingly, the miR-223 expression level of PBMCs was significantly increased after acute gout remission in comparison with that measured after gout flares in 30 paired cases (*p* < 0.001, Figure 1B). These results indicated that miR-223 might be involved in regulating the spontaneous resolution of acute gouty inflammation.

### Altered Expression of miR-223, the NLRP3 Inflammasome and Cytokines in MSU-Induced RAW264.7 Murine Macrophage Inflammation

**FIGURE 2:**
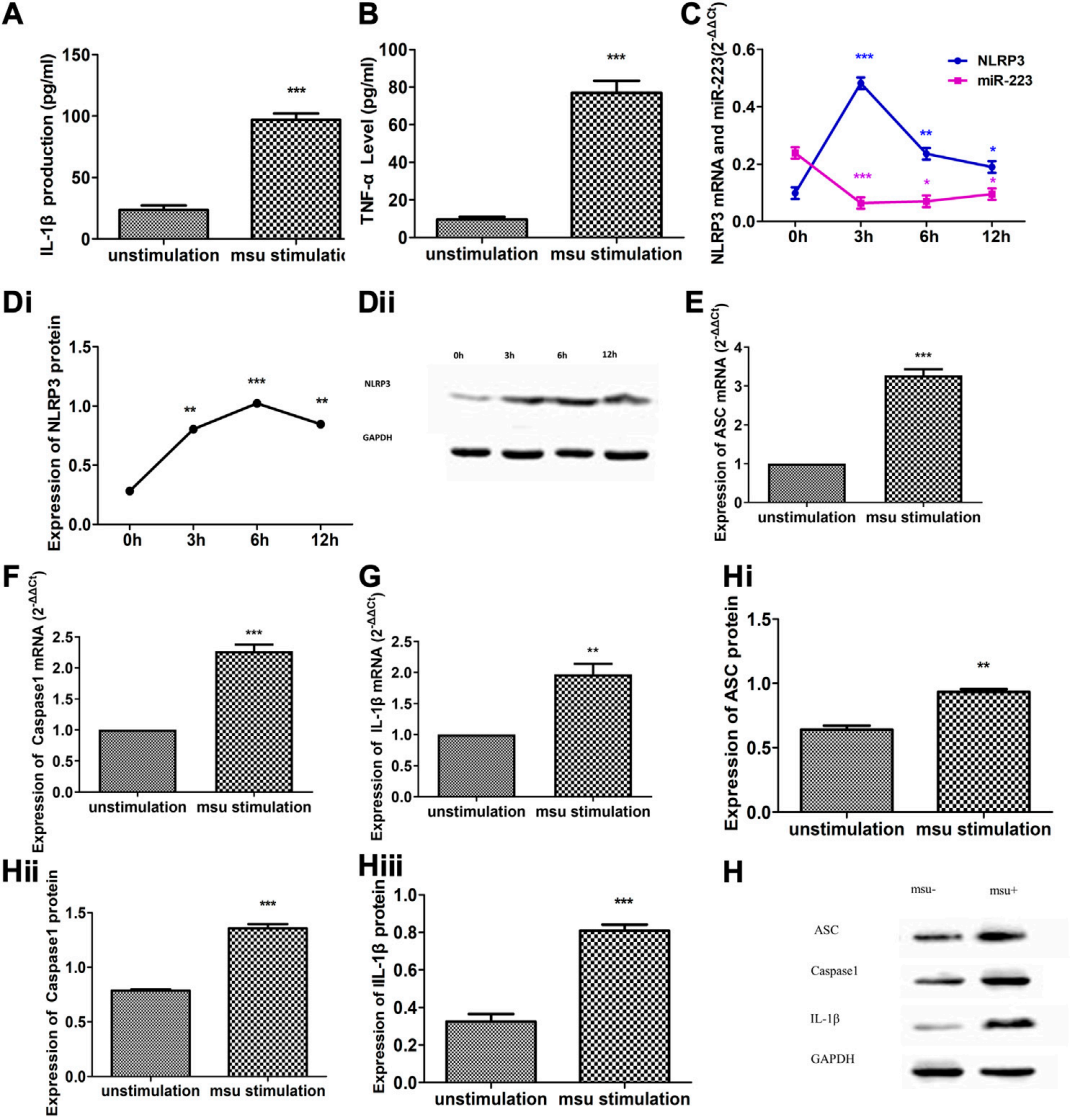
Altered expression of miR-223, NLRP3 inflammasome and cytokines in MSU crystal-induced RAW264.7 murine macrophage inflammation. **(A, B)** IL-1β and TNF-α levels were dramatically increased in cultural supernatants after RAW264.7 macrophages were treated with MSU for 6 h (*n* = 5). **(C)** Altered expression of NLRP3 mRNA and miR-223 upon stimulation of RAW264.7 cells in comparison with that of unstimulated control cultures (*n* = 5). NLRP3 mRNA up-regulation of approximately 4.88-fold after MSU stimulation for 3 h, 2.39-fold after 6 h, and 1.92-fold after 12 h. MiR-223 down-regulation of approximately 3.71-fold after MSU stimulation for 3 h, 3.39-fold after 6 h, and 2.50-fold after 12 h. **(Di, Dii)** Altered expression of NLRP3 protein upon stimulation of RAW264.7 cells in comparison with that of unstimulated control cultures (*n* = 5). NLRP3 protein up-regulation of approximately 2.85-fold after MSU stimulation for 3 h, 3.62-fold after 6 h, and 2.99-fold after 12 h. **(E–H)** Altered expression of ASC, caspase-1 and IL-1β mRNA and protein in RAW264.7 macrophages stimulated with MSU for 6 h *p < 0.05, **p < 0.01.

Treatment of RAW264.7 macrophages with crystalline MSU for 12 h altered the expression levels of cytokines, the NLRP3 inflammasome and miR-223 (Figure 2). The abundance of IL-1β and TNF-α in the culture supernatants was significantly increased following treatment with MSU (*p* < 0.001, respectively; Figures 2A,B). After MSU stimulation, a significant reduction in miR-223 expression was observed in RAW264.7 macrophages together with significantly increased expression of NLRP3 (*p* < 0.05, respectively; Figures 2C, Di,ii). The greatest changes in the abundance of miR-223 and NLRP3 mRNA were observed 3 h after MSU treatment (Figure 2C), while the peak NLRP3 protein expression occurred 6 h after MSU treatment (Figures 2Di,ii). In addition, the mRNA and protein levels of ASC, caspase1 and IL-1β were significantly increased in RAW264.7 macrophages after MSU treatment (Figures 2E–H). These results suggest that MSU can activate the NLRP3 inflammasome and induce inflammation. Moreover, these findings suggest that miR-223 expression is oppositely regulated in comparison with cytokines and the NLRP3 inflammasome following MSU-induced acute inflammation.

### Effect of miR-223 on Cytokine Secretion From RAW264.7 Macrophages Treated With MSU

**FIGURE 3:**
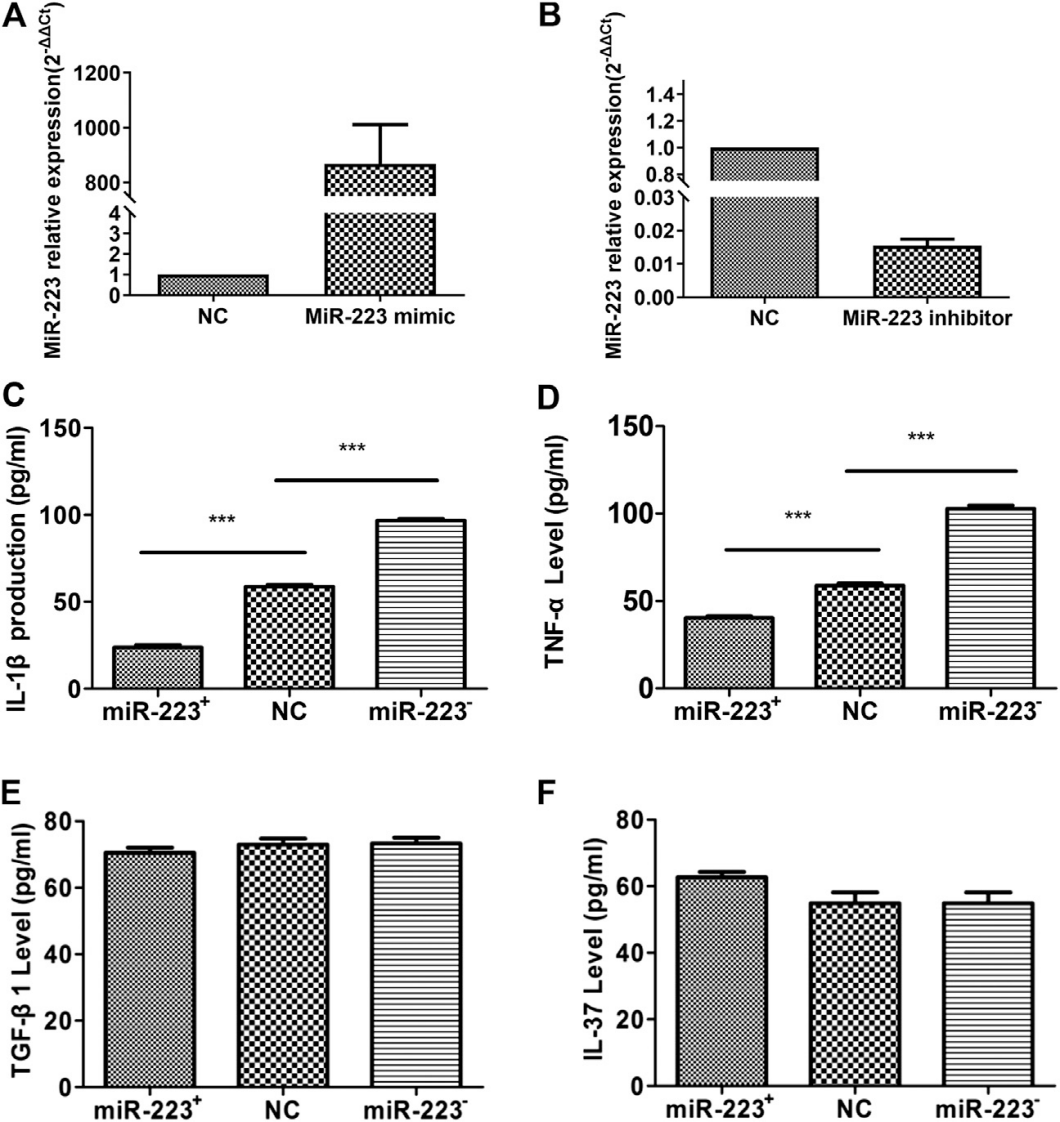
Effect of miR-223 on the secretion of cytokines from RAW264.7 murine macrophages treated with MSU. **(A)** Transfection of miR-223 mimics (100 nM) for 24 h increased miR-223 expression approximately 1000-fold in RAW264.7 macrophages. **(B)** Transfection of miR-223 inhibitors (100 nM) for 24 h significantly decreased miR-223 expression by at least 80% in RAW264.7 macrophages. **(C, D)** Increased miR-223 abundance suppressed production of IL-1β and TNF-α (*n* = 5, t = −18.67, −11.214), while decreased miR-223 abundance enhanced IL-1β and TNF-α production compared to that of the NC cells (*n* = 5, t = 24.45,19.07), ***p < 0.001. **(E, F)** Secretion of TGF-β1 and IL-37 was not affected by increased (*n* = 5, t = 1.041, 0.001; *p* > 0.05, respectively) or decreased (*n* = 5, t = 0.146, 2.126; *p* > 0.05, respectively) expression of miR-223 in RAW264.7 cells in comparison with NC cells. The cells were transfected with 100 nM of a miR-223 mimic or miR-223 inhibitor for 24 h, then stimulated with 200 μg/ml MSU for 12 h, and finally evaluated of TNFa, IL-1, TGF-β1 and IL-37 in supernatant culture.

To determine the effect of miR-223 on cytokine secretion from RAW264.7 macrophages treated with MSU, we transfected RAW264.7 macrophages with either a miR-223 mimic or a miR-223 inhibitor, which increased or decreased the cellular levels of mature miR-223, respectively. RT-qPCR was carried out to validate miR-223 up-regulation/down-regulation in RAW264.7 macrophages (Figures 3A,B). Transfection with the miR-223 mimic or inhibitor for 24 h, and then treated RAW264.7 macrophages with MSU for 12 h. The expression levels of IL-1β, TNF-α, IL-37, and TGF-β1 in the culture supernatants were measured using ELISA. Following treatment with the miR-223 mimic, concentrations of IL-1β and TNF-α were significantly decreased (Figures 3C,D), while IL-37 and TGF-β levels were not significantly changed (Figures 3E,F). When endogenous expression of miR-223 in RAW264.7 macrophages was silenced, IL-1β and TNF-α levels were significantly increased (Figures 3C,D), while IL-37 and TGF-β1 were not influenced (Figures 3E,F). These results suggest that miR-223 negatively regulates IL-1β and TNF-α production during MSU-induced inflammation, whereas IL-37 and TGF-β1 might not be regulated by miR-223.

### No Effect on RAW264.7 Macrophage Apoptosis

**FIGURE 4:**
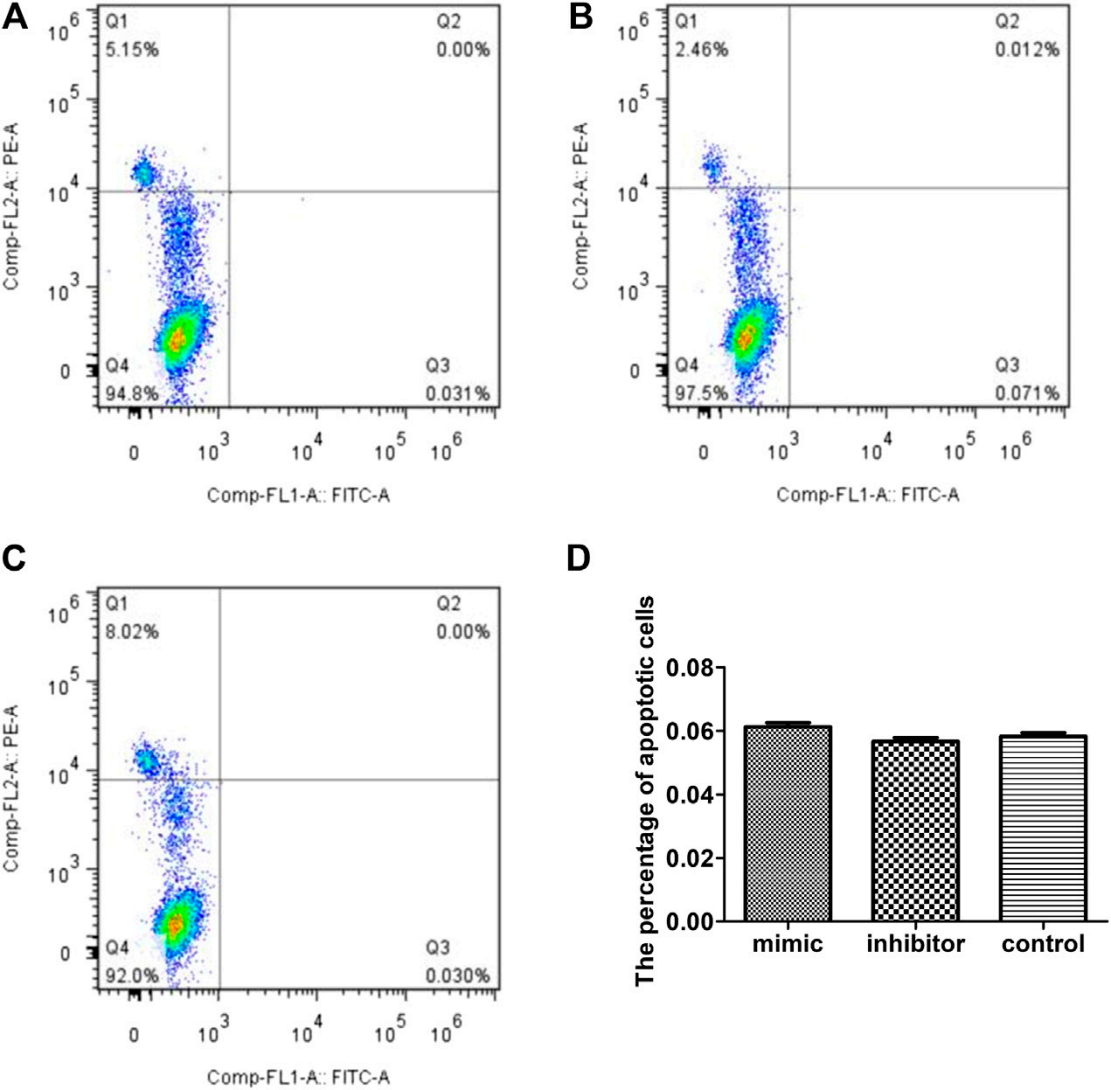
Effect of miR-223 on RAW264.7 macrophage apoptosis. Effect of miR-223 mimics **(A)**, miR-223 inhibitors **(B)** and the scrambled control **(C)** on RAW264.7 macrophage apoptosis. Representative results are shown. **(D)** Comparison of the percentage of apoptotic cells among the three groups. The percentage of apoptotic cells (annexin V-positive/PI-negative) was 0.061 om0.028%, 0.056 .00.027% and 0.058 020.026%, respectively, in the miR-223 mimic group, miR-223 inhibitor group and scrambled control group. The differences among the groups were not statistically significant (*n* = 5, *p* > 0.05).

To test whether the inhibitory effect of miR-223 on the survival of RAW264.7 macrophages treated with MSU was associated with apoptosis, the percentage of apoptotic cells was measured using annexin V/PI staining. The percentage of PI/annexin V+ cells after transfection with the miR-223 mimic was 0.061 ± 0.028%, which was similar to that observed following transfection with the miR-223 inhibitor and that of the scrambled control group (*p* > 0.05, Figure 4).

### Effect of miR-223 on NLRP3 Inflammasome Expression in RAW264.7 Macrophages Treated With MSU

**FIGURE 5:**
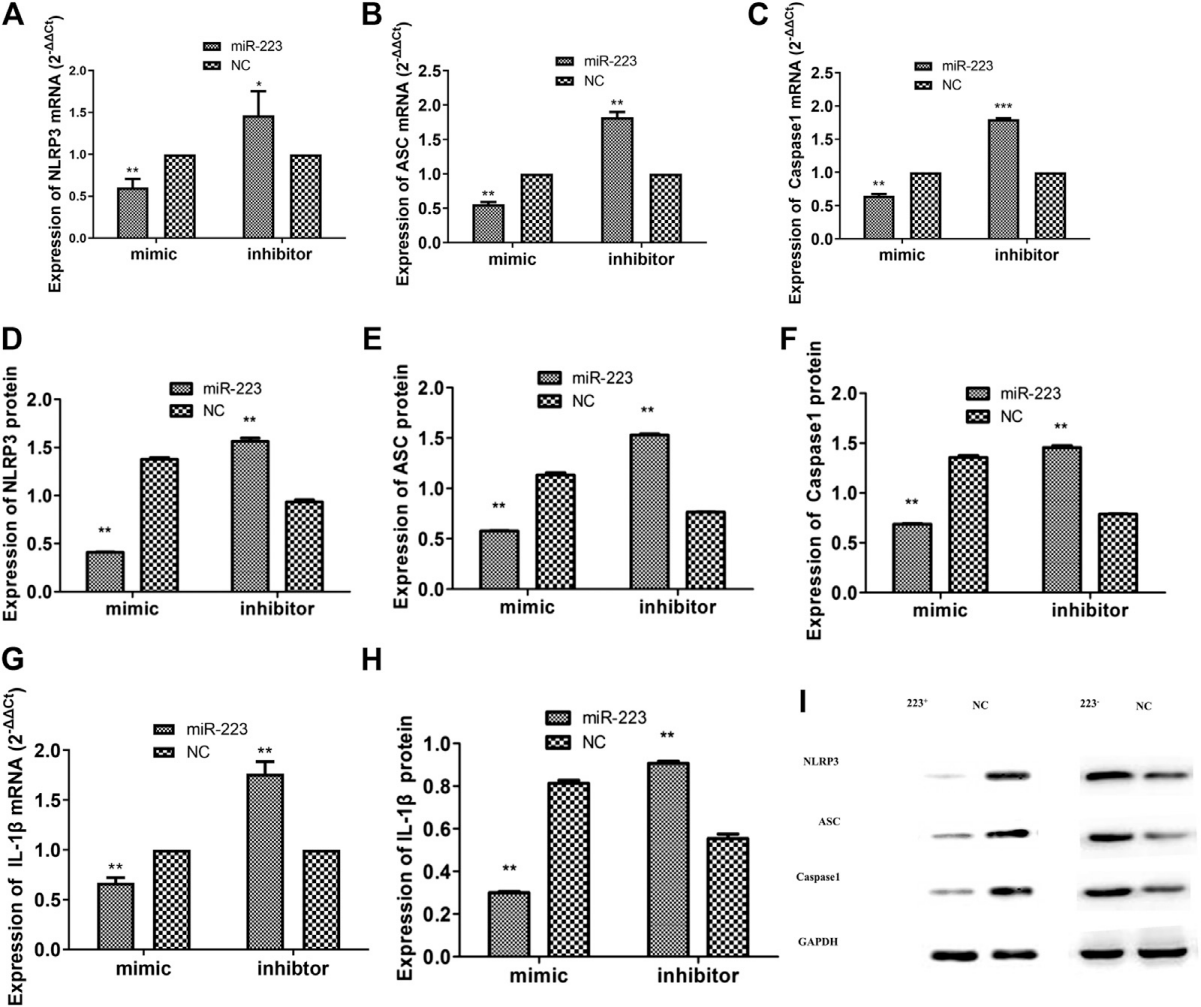
Effect of miR-223 on expression of the NLRP3 inflammasome and IL-1β in RAW264.7 macrophages treated with MSU. Over-expression of miR-223 significantly decreased the mRNA and protein expression levels of the NLRP3 inflammasome (NLRP3, ASC and caspase-1) and IL-1β in comparison with the miRNA negative controls in RAW264.7 macrophages. In contrast, miR-223 inhibitors significantly increased the mRNA and protein expression levels of the NLRP3 inflammasome and IL-1β in comparison with the miRNA negative controls in RAW264.7 macrophages (*p < 0.05, **p < 0.01).

The experiments described above showed that MSU activated the NLRP3 inflammasome (NLRP3, ASC, and caspase-1) as an endogenous danger signal. To investigate whether miR-223 regulates the NLRP3 inflammasome in MSU-induced inflammation, RAW264.7 macrophages transfected with the miR-223 mimic/inhibitor described above were stimulated with MSU, after which the mRNA and protein levels of the NLRP3 inflammasome (NLRP3, ASC, and caspase-1) were determined using RT-qPCR and western blotting, respectively. NLRP3 transcripts were previously identified as targets of miR-223 using a luciferase assay (Bauernfeind et al., 2012; Haneklaus et al., 2012). The NLRP3 pathway plays a vital role in the pathophysiology of acute gout. Pro-IL-1β is cleaved to its mature form by caspase-1, and the NLRP3 inflammasome is essential for caspase-1 activation. Even though the other two components (ASC and caspase-1) of the inflammasome are not direct targets of miR-223, we assessed the mRNA and protein expression levels of all components of the NLRP3 inflammasome (NLRP3, ASC, and caspase-1) in MSU-treated RAW264.7 macrophages. Surprisingly, we found that miR-223 over-expression significantly decreased the mRNA and protein levels of all components of the NLRP3 inflammasome (NLRP3, ASC, caspase-1) (Figure 5). In addition, the miR-223 inhibitor significantly increased the protein and mRNA levels of the components of the NLRP3 inflammasome (Figure 5). These results suggest that miR-223 could negatively regulate NLRP3 expression and inhibit the activity of the NLRP3 inflammasome.

## Discussion

To our knowledge, this study is the first to suggest that miR-223 might negatively regulate acute gouty inflammation by suppressing production of IL-1β and TNF-α by targeting the NLRP3 inflammasome, and not by influencing IL-37 and TGF-β1.

Gout is a typical innate immune response triggered by MSU. As an “endogenous danger signal,” MSU can induce the onset of gout inflammation and cause immune abnormality. Spontaneous remission of acute inflammation is one of the clinical characteristics of gout that differentiate it from other auto-inflammatory diseases. Studies (Lioté et al., 1996; Steiger and Harper, 2014; Zeng et al., 2016) suggest that IL-37 and TGF-β1 might participate in self-limiting of gouty inflammation. To date, the concrete molecular mechanism of gout spontaneous remission is unclear. MiRs are endogenous non-coding small RNAs that can regulate the expression of mRNA after transcription. They combine with the 3′-untranslated region of the target mRNAs to form RNA-induced silencing complexs (RISC), which then degrade the target mRNAs or inhibit the translation of the target mRNAs, and exert corresponding biological effects (Bartel, 2004). In addition, one miR can regulate multiple mRNAs, and one mRNA can also be regulated by multiple miRs (Bartel, 2004). Previous studies have demonstrated that miRs can negatively regulate inflammation in some auto-inflammatory diseases (Henshall et al., 2016), such as miR-146a, miR-155, miR-302b, miR-488, miR-920, and miR-221–5p have been shown to limit MSU-induced inflammation (Taganov et al., 2006; Jin et al., 2014; Dalbeth et al., 2015; Zhou et al., 2017; Ma et al., 2018; Li et al., 2020). However, it is unclear whether miR-223 participates in regulating gouty inflammation.

Gout is one of the most common arthritis. In United Kingdom and United States studies, the incidence of gout varies from 0.30 per 1,000 person-years in the 1970s to 2.68 per 1,000 person-years in the 2000s (Kuo et al., 2015), and the incidence rate in male is far higher than female (Tang et al., 2021). In this study, almost all samples we collected from patients with gout were men. In order to reduce the bias of the data, only male patients were chosen as the subjects included. Compared with intercritical gout patients and healthy controls, the expression of miR-223 in the PBMCs of patients with acute gout was significantly decreased. Our analysis revealed that miR-223 expression was significantly increased after gout resolution. In addition, higher leukocyte and neutrophil counts were observed in patients with acute gout, whereas negative correlations were observed between miR-223 expression and leukocyte and neutrophil counts. These results suggest that miR-223 might be involved in negatively regulating gouty inflammation. Interestingly, significantly increased production of IL-1β and TNF-α was observed after miR-223 down-regulation, whereas decreased levels of IL-1β and TNF-α were observed after miR-223 over-expression, while production of IL-37 and TGF-β1 remained unchanged whether miR-223 expression was increased or decreased. These results suggest that miR-223 could negatively regulate IL-1β and TNF-α production, but that it does not influence IL-37 and TGF-β1. IL-1β plays a critical role in acute gouty inflammation, while TGF-β1 and IL-37 play important roles in limiting inflammation (Lioté et al., 1996; Steiger and Harper, 2014; Zeng et al., 2016; Renaudin et al., 2020). Our findings suggest that miR-223 might be involved in the pathogenesis of gouty remission by suppressing production of IL-1β and TNF-α, but not by regulating IL-37 and TGF-β1.

**FIGURE 6:**
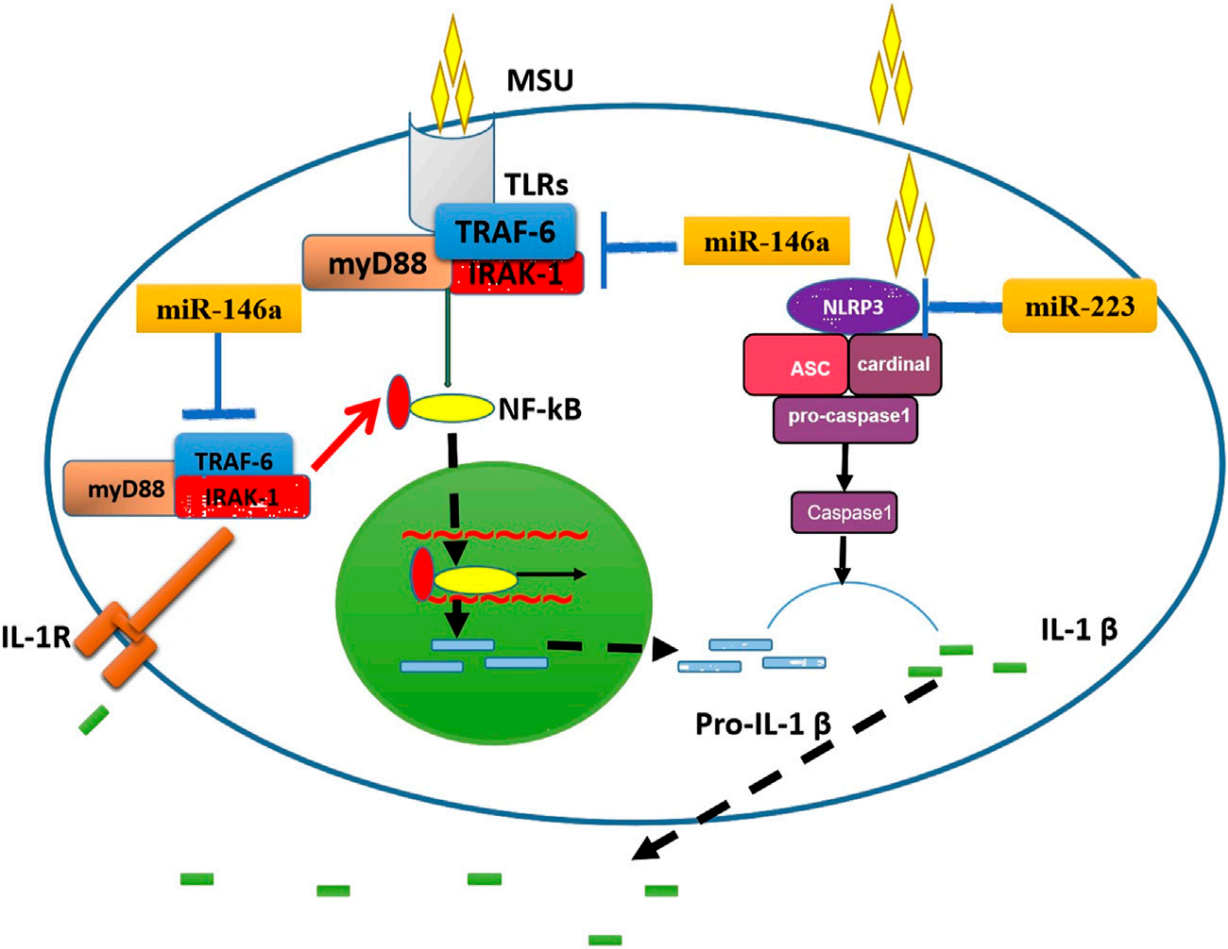
The potential mechanisms by which miR-146a and miR-223 influence gouty arthritis. On the left, the schematic shows how miR-146a might decrease IL-1β production by inhibition of TRAF6 and IRAK1 function. On the right, the schematic shows how miR-223 reduces IL-1β production by inhibiting NLRP3 function. ASC, apoptosis-associated speck-like protein; IL-1β, interleukin-1β; IL-1R, IL-1 Receptor; IRAK1, IL-1 receptor-associated kinase 1; MSU, monosodium urate; NLRP3, NACHT-LRR-PYD-containing protein; Pro-IL-1β, precursor of IL-1β; TLR, toll-like receptor; TRAF6, TNF receptor-associated factor 6.

The occurrence of MSU-mediated gout inflammation is a complex process, including the phagocytosis of autoimmune cells, the negative regulation of inflammatory mediators such as NLRP3 inflammasomes, Toll-like receptors and IL-1β, and the formation of aggregated neutrophil extracellular traps, etc (Qing et al., 2014; Dalbeth et al., 2016). IL-1β is an important inflammatory mediator of gouty arthritis, which is mainly produced through the TLR4-NF-κB and NLRP3 inflammasome signaling pathways (Garcia-Martinez et al., 2015). NF-κB is activated by TLR4 to promote the production of pro-IL-1β, which is then processed by NLRP3 inflammasomes from pro-IL-1β into mature IL-1β, and finally IL-1β is released outside the cell. MSU can be used as an “endogenous danger signal” to activate TLR4 and NLRP3 inflammasomes in a similar way to pathogen-associated molecular patterns (PAMPs), causing a series of inflammatory cascades (Qing et al., 2014; Dalbeth et al., 2016). (Figure 6). In particular, the research of Taganov KD et al. (Taganov et al., 2006) have shown that miR-146 can regulate the TLR4 pathway: IL-1 receptor-associated kinase 1 (IRAK1) and TNF receptor-associated factor 6 (TRAF6) are the key receptor kinases downstream of the TLR4 pathway and are also the target molecules of miR-146a. When the TLR4 pathway is activated, on the one hand, these two kinases promote the downward transmission of inflammatory signals by inducing the activation of the NF-kB pathway; On the other hand, miR-146a can specifically bind to the 3′-untranslated regions of IRAK1 and TRAF6, and down-regulate the levels of IRAK1 and TRAF6 through a negative feedback mechanism, thereby negatively regulating the inflammatory response. Our previous study (Zhang et al., 2018) used miR-146a knockout mice to test miR-146a function in a MSU-induced gouty arthritis model, and the results indicated that the lack of miR-146a enhances the inflammation level of gouty arthritis via upregulation of TRAK6, IRAK-1, and the NALP3 inflammasome function. It further confirmed that miR-146a can inhibit inflammation and is consistent with Taganov KD's research results (Taganov et al., 2006).

The NLRP3 inflammasome plays a key role in the pathogenesis of gouty inflammation (Martinon et al., 2006; So and Martinon, 2017; Renaudin et al., 2020). One study (Martinon et al., 2006) found that NLRP3 is a type of cytoplasmic pattern recognition receptor, and the NLRP3 inflammasome is an inflammatory complex formed by NLRP3, ASC, and caspase-1. The leucine-rich region located at the C-terminus of the NLRP3 molecule can recognize the endogenous danger signals caused by MSU, and then MSU combines with NLRP3 to promote the activation of NLRP3 inflammasomes and drive caspase-1 to process pro-IL-1β into mature IL-1β, which leads to gout flare (Martinon et al., 2006). NLRP3 components were previously identified as targets of miR-223 using luciferase assays, and NLRP3 has been shown to directly increase IL-1β production during innate immune responses (Bauernfeind et al., 2012; Haneklaus et al., 2012). However, there is no report that miR-223 participates in the regulation of acute gout inflammation by affecting NLRP3. These findings prompted us to explore whether miR-223 regulates IL-1β mediated gouty inflammation by targeting NLRP3 using RAW264.7 macrophages in a model of MSU-induced inflammation. We found that miR-223 over-expression reduced NLRP3 mRNA and protein levels in RAW264.7 macrophages, whereas miR-223 under-expression increased NLRP3 mRNA and protein levels. These results strongly suggest that miR-223 can negatively regulate gouty inflammation by targeting NLRP3. Pro-IL-1β is cleaved to its mature form by caspase-1, and the NLRP3 inflammasome is essential for caspase-1 activation. Although ASC and caspase-1 are not direct targets of miR-223, we assessed mRNA and protein expression of ASC and caspase-1 in MSU-treated RAW264.7 macrophages. Interestingly, we found that the mRNA and protein levels of ASC and caspase-1 were dramatically decreased when miR-223 was over-expressed, while they were significantly increased when miR-223 was under-expressed. These results suggest the possibility that miR-223 indirectly targets ASC or caspase-1 to suppress the activation of IL-1β. TNF-α production might be regulated indirectly by the NLRP3 inflammasome and miR-223, or currently unknown regulatory mechanisms may exist to promote this effect.

Therefore, we speculate that there may be negative feedback regulation in patients with gout, when MSU crystals act on the NLRP3 inflammasome to cause acute inflammation. The targeted binding of miR-223 to NLRP3 mRNA increases, which inhibits the activity of NLRP3 inflammasomes and reduces the release of IL-1β, thereby reducing acute inflammation, which also better explains the phenomenon that gout patients can spontaneously relieve within 7–14 days after an acute gout flare (Dalbeth et al., 2016). However, it has been recognized that one miR can regulate multiple mRNAs, and one mRNA can also be regulated by multiple miRs. Therefore, miR-223 may not be the only molecule that explains this phenomenon. Other miRs such as miR-146a, miR- 155, miR-302b, miR-488, miR-920, and miR-221-5p may be involved in the regulation of the inflammatory response process of gout, and the specific mechanism is still unclear. Of course, we must admit the fact that miR-223 mimic induced a 1000-fold overexpression or underexpression of the target, which does not exist *in vivo*. However, in the *in vitro* test state, only a higher transient transfection multiple can maintain the high or low expression of miR-223 in the cell model to study the biological functions of miR-223. For these, we need to do more work next, Such as establishing animal models for *in vivo* further research. Besides, whether the expression levels of miR-223 and other inflammatory mediators in the synovial fluid of gout patients are consistent with those in PBMCs, this will also be the direction of our next research.

## Conclusion

miR-223 has been identified as an important regulator of inflammatory and immune responses that is implicated in several auto-inflammatory disorders. However, the expression status of miR-223 in gout patients and the mechanisms through which miR-223 regulates gouty inflammation remain unknown. Here, we measured miR-223 expression in acute and intercritical gout patients, after which we used RAW264.7 macrophages transfected with a miR-223 mimic or inhibitor to assess the function of miR-223 in MSU-induced gouty inflammation. Our findings suggest that miR-223 provides negative feedback regulation of gouty inflammation development by suppressing production of IL-1β and TNF-α, but not by regulating IL-37 and TGF-β1, and that miR-223 regulates cytokine production by targeting the NLRP3 inflammasome. These findings provide novel insight into the regulatory role of miR-223 in the spontaneous resolution of acute gouty inflammation. Finally, these results suggest that targeting miR-223 in macrophages might be an effective therapeutic strategy for the treatment of gout flare.

## Data Availability

The original contributions presented in the study are included in the article/Supplementary Material, further inquiries can be directed to the corresponding author.
